# A Comprehensive Numerical Model for Simulating Fluid Transport in Nanopores

**DOI:** 10.1038/srep40507

**Published:** 2017-01-16

**Authors:** Yuan Zhang, Wei Yu, Kamy Sepehrnoori, Yuan Di

**Affiliations:** 1 College of Engineering, Peking University, Beijing, 100871, China; 2 Department of Petroleum Engineering, Texas A & M University, Collage Station, TX, 77843, USA; 3 Department of Petroleum and Geosystems Engineering, University of Texas at Austin, Austin, TX, 78712, USA

## Abstract

Since a large amount of nanopores exist in tight oil reservoirs, fluid transport in nanopores is complex due to large capillary pressure. Recent studies only focus on the effect of nanopore confinement on single-well performance with simple planar fractures in tight oil reservoirs. Its impacts on multi-well performance with complex fracture geometries have not been reported. In this study, a numerical model was developed to investigate the effect of confined phase behavior on cumulative oil and gas production of four horizontal wells with different fracture geometries. Its pore sizes were divided into five regions based on nanopore size distribution. Then, fluid properties were evaluated under different levels of capillary pressure using Peng-Robinson equation of state. Afterwards, an efficient approach of Embedded Discrete Fracture Model (EDFM) was applied to explicitly model hydraulic and natural fractures in the reservoirs. Finally, three fracture geometries, i.e. non-planar hydraulic fractures, non-planar hydraulic fractures with one set natural fractures, and non-planar hydraulic fractures with two sets natural fractures, are evaluated. The multi-well performance with confined phase behavior is analyzed with permeabilities of 0.01 md and 0.1 md. This work improves the analysis of capillarity effect on multi-well performance with complex fracture geometries in tight oil reservoirs.

Tight reservoirs make significant contributions to the oil and gas productions in recent years. These reservoirs are unique because of the low permeability, which requires multiple horizontal wells and multistage hydraulic fractures for economic production^1^. Permeability is the intrinsic property of rocks, which measures the ability of rocks to allow liquids or gases to flow through it. Permeability is typically expressed in units of millidarcy (md), and a large value indicates the liquids or gases are highly permeable through rocks. Since the natural fractures are generally distributed in the formation, the combination of horizontal drilling and hydraulic fracturing is an efficient approach to generate a large contact area between the wellbore and low permeability formation. The actual fracturing process often creates complex fracture geometries in tight reservoirs due to the pre-existing natural fractures^2^,^3^.

High capillary pressure due to nanopores in tight reservoirs impacts the fluid phase behavior and long-term estimated ultimate recovery (EUR) prediction in unconventional reservoirs^4^,^5^. Theoretical work has shown that the bubble-point pressure decreases due to the reduction of pore radius^6^,^7^,^8^,^9^. The further the reservoir condition is from the critical point, the larger the reduction of bubble-point pressure. Since traditional Pressure-Volume-Temperature (PVT) analysis failed to calculate the phase behavior with the capillary pressure effect, recent studies have focused on developing new algorithms to evaluate its effect on fluild properties and well performance in unconventional reservoirs. Du and Chu^10^ used a commercial reservoir simulator to investigate the influence of PVT variations on single-well performance in the Bakken formation. They discussed the confined PVT properties in two permeability scenarios, however, the pore size distribution and fracture networks were not included in their work. Alternately, Wang *et al*.^11^ incorporated the capillary pressure effect and pore space compaction in a compositional reservoir simulator to evaluate their effects on production. They considered the change of porosity and permeability with effective stress, but no discrete fractures were considered in their simulations. On the other hand, Nojabaei *et al*.^12^ used a compositionally-extended black oil model to incorporate the capillarity effect on phase behavior. The pore size distribution was considered in their model, but the hydraulic fractures were not included for simulation of single-well performance. Rezaveisi *et al*.^13^ implemented the capillarity equilibrium in an in-house compositional reservoir simulator to forecast the well performance. They compared oil recoveries of tight oil and gas condensate cases, but complex fractures geometries were not investigated. Furthermore, Melero *et al*.^14^ investigated the effect of fracture complexity on the well performance of CO_2_-EOR in tight oil reservoirs. However, the capillary pressure effect was not taken into account. While Wu and Zhang^15^ discussed the gas transport in nanopores by molecular dynamic simulation; Wang *et al*.^16^ used the molecular dynamics model to reproduce the experimental contact angle and calibrate the parameters with nanopore confinement, but neither considered the fractures in the formations. Additionally, Siripatrachai *et al*.^17^ developed a compositional reservoir simulator incorporating the capillary pressure effect on phase behavior with several discrete hydraulic fractures. However, the effect of complex natural fractures in simulation of multi-well performance was not examined.

The objective of this study is to investigate the capillary pressure effect on performance of multiple wells with different fracture geometries. Newton fluid is considered and the governing equations for the oil and gas transport are applied in this work. Embedded Discrete Fracture Model (EDFM) is utilized to efficiently handle the complex fractures. First of all, EDFM was verified to approach for bi-wing hydraulic fractures against Local Grid Refinement (LGR) method, which can accurately simulate the oil and gas flow from shale matrix to simple fractures^18^. Subsequently, the verified methodology was used to build a numerical reservoir model including four horizontal wells to simulate the capillary pressure effect on well performance with three complex fracture geometries. Two permeabilities of 0.01 md and 0.1 md are taken into account. This work improves the analysis of multi-well performance with complex fracture geometries in tight oil reservoirs.

## Results

### Base case

**Figure 1:**
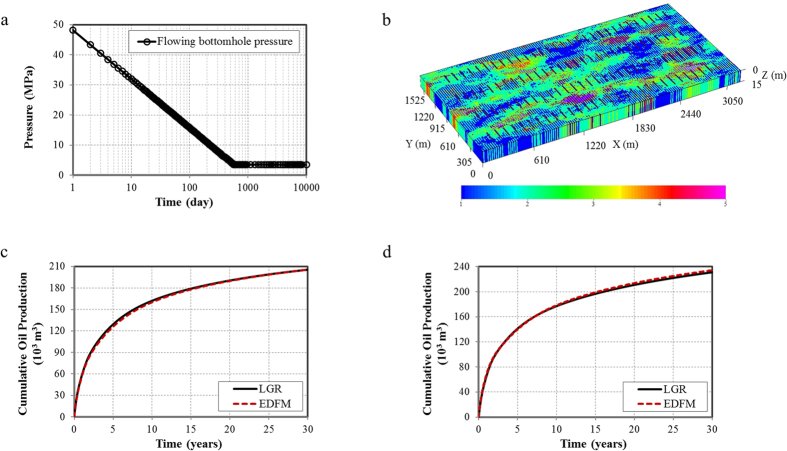
Verification of EDFM approach. (**a**) Flowing bottomhole pressure for simulation of Middle Bakken tight oil reservoirs; (**b**) Nanopore size distribution in the reservoir model. (Color bar of 1–5 respresents five different PVT regions: less than 10 nm, 10–20 nm, 20–30 nm, 30–50 nm, and larger than 50 nm); (**c**) Comparison of cumulative oil production for a 30-year period without the capillary pressure effect using EDFM and LGR approach; (**d**) Comparison of cumulative oil production for a 30-year period without the capillary pressure effect using EDFM and LGR approach.

The Bakken formation is located in the Williston Basin. It consists of Upper and Lower Bakken Shales, Middle Bakken, and Three Forks. The Middle Bakken region, which is the main pay zone, has an estimated average oil resource of 3.65 billion barrels^19^,^20^. In the development of Middle Bakken formation, four horizontal wells in a reservoir size of 1 mile × 2 miles are often drilled with multistage hydraulic fractures, which permit more connected fracture networks for production^21^. Therefore, a base reservoir model is set up, including four horizontal wells with 30 planar hydraulic fractures for each well (Supplementary Figure 1), which is more reliable than modeling a single well. The reservoir size is 3,219 m × 1,609 m × 15 m, corresponding to length, width, and thickness, respectively. It consists of 264 grids in *x* direction, 132 grids in *y* direction, and 1 grid in *z* direction. The horizontal wells with lateral length of 2,829 m are also incorporated in the model. The fractures in the neighboring wells are in a zipper pattern, with fracture spacing of 97 m and fracture half-length of 79 m. The initial reservoir pressure is 51.7 MPa, and initial water saturation is 49%. The compositions and critical properties has been described previously^19^. Matrix permeability is 0.071 md, and matrix porosity is 5.6%. Total compressibility is 1.5 × 10^−15^ MPa^−1^ and fracture conductivity is 15 md-m. The reservoir temperature is 116 °C. The water-oil and liquid-gas relative permeability curves are from the previous study^22^, which were obtained from history matching from a production well in the Middle Bakken formation. It is noted that the oil and gas properties, rock properties, and fracture properties are within the reasonable range of typical oil properties reported in the literature^19^,^23^,^24^,^25^. In real field production, the bottomhole pressure does not decrease rapidly, but gradually from a larger value to a smaller value within a certain period. In order to mimic the actual field observation, bottomhole pressure was set to decrease from 48.3 to 3.5 MPa around one and a half years, and remains 3.5 MPa afterwards until the end of production, as shown in Fig. 1(a). The low and high values of flowing bottomhole pressure are given based on the field observation.

### Verification of Embedded Discrete Fracture Model (EDFM)

The performance of base case was evaluated to verify the EDFM approach against LGR approach with and without the capillary pressure effect. As the aforementioned discussion, EDFM approach is easier for handling the complex fracture networks and its computational speed is several times faster than the LGR approach when modeling complex fracture geometries^26^.

In order to consider the capillary pressure effect, the raw data of Mercury Injection Capillary Pressure (MICP) test and the nanopore size distribution were used for the Middle Bakken formation^23^,^24^,^26^,^27^, and the nanopore sizes were divided into five different regions: less than 10 nm, 10–20 nm, 20–30 nm, 30–50 nm, and bulk (pore sizes larger than 50 nm). It should be mentioned that different rocks need different patterns and the division mainly depends on the actual pore size distribution of the tight rock. The sensitivity studies were then performed to identify the computation time and accuracy of different patterns. Finally, distribution into five regions is found to better maintain the balance of accuracy and computation speed. Figure 1(b) displays the distribution of five different regions in the base reservoir model when the capillary pressure effect is considered.

The comparison of cumulative oil production of four horizontal wells for 30 years with and without the capillary pressure effect using LGR and EDFM approach is presented in Fig. 1(c) and (d). The cumulative oil or gas production is defined as the total amount of oil and gas production from an oil reservoir of a particular time. It is based on the mass balance equation and can be calculated by multiplying the amount of production by rate. The calculation of cumulative production can be found in the supplementary information. As shown, a good agreement is obtained, indicating that the EDFM approach can well simulate the well performance with hydraulic fractures in tight oil reservoirs.

It should be noted that in the real case studies, there are complex non-planar hydraulic fractures with natural fractures. The LGR method is challenging to deal with such complex fracture geometries. The EDFM approach can be applied to effectively capture the complex fracture geometries and simulate their effects on oil and gas transport phenomenon in fractured porous media. Although the simulation production results were not compared with the actual field production data, which are difficult to obtain now, the simulations and prediction results are reasonable, for that the reservoir, fracture, and fluid properties used in the case studies are based on the experimental data in the Bakken tight oil reservoir^22^,^23^,^24^,^25^. In the future, great efforts will be made to further investigate the comparisons with the actual field production data.

### Effect of different fracture geometries on well performance

Investigation of the effect of different fracture geometries on well performance were performed in this section. Since the non-planar hydraulic fractured cases with natural fractures represent the realistic fracture networks in tight reservoirs, the simulation tests for four different fracture geometries for the following cases:

- Case 1: Non-planar hydraulic fractures.
- Case 2: Non-planar hydraulic fractures with 500 natural fractures (NF) in one set.
- Case 3: Non-planar hydraulic fractures with 1,000 natural fractures (NF) in one set.
- Case 4: Non-planar hydraulic fractures with 1,000 natural fractures (NF) in two sets.

**Figure 2:**
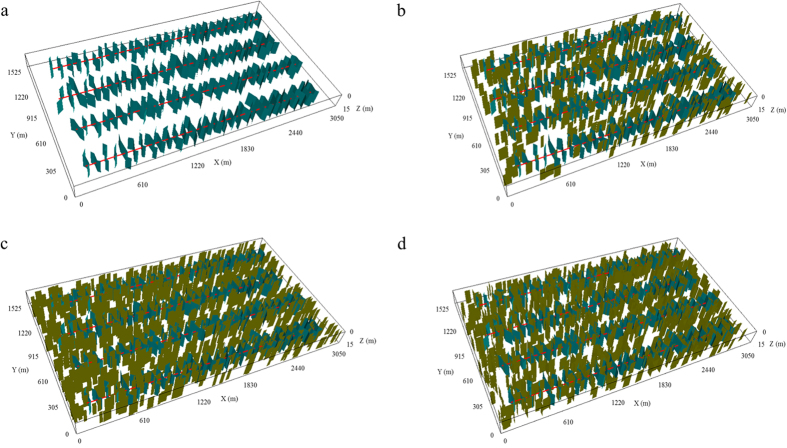
Fracture distribution for four different models considering hydraulic and natural fractures. (**a**) Non-planar hydraulic fractures; (**b**) Non-planar hydraulic fractures with 500 NF in one set; (**c**) Non-planar hydraulic fractures with 1,000 NF in one set; (**d**) Non-planar hydraulic fractures with 1,000 NF in two sets.

Fracture geometries for four cases are depicted in Fig. 2. The fracture half-length varies from 45.7 to 137.2 m. Natural fractures is normally distributed in the models based on the assumption that their orientations are parallel to the horizontal wellbore. The angles along the *x* direction for one set of natural fractures are between 0–10 deg and 90–100 deg for another set. The length of natural fractures ranges from 30.5 to 91.4 m and the nature fracture conductivity is 1.5 md-m. The hydraulic fracture conductivity is 15 md-m.

**Figure 3:**
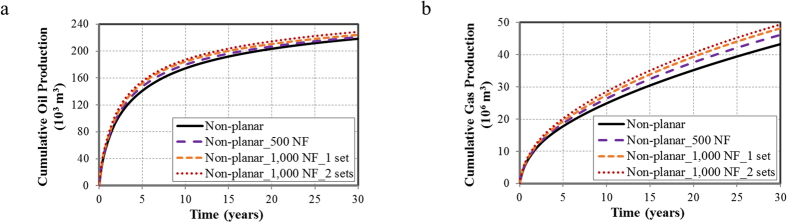
Well performance for a 30-year period for different fracture geometries. (**a**) Cumulative oil production; (**b**) Cumulative gas production.

The simulation tests for four cases investigate the impacts of different fracture geometries and the contribution of the natural fractures on well performance. The simulation results for four cases are presented in Fig. 3.

**Figure 4:**
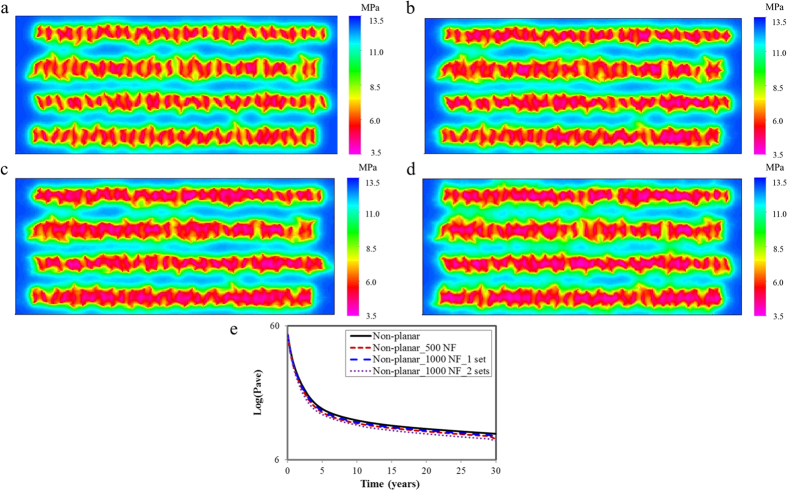
Pressure distribution for four cases at the end of 30 years. (**a**) Non-planar hydraulic fractures; (**b**) Non-planar hydraulic fractures with 500 NF; (**c**) Non-planar hydraulic fractures with 1,000 NF in one set; (**d**) Non-planar hydraulic fractures with 1,000 NF in two sets; (**e**) Difference of average pressure of four cases.

As illustrated in Fig. 3, the cumulative oil and gas production increase as the fracture geometries become complex. When there are 500 natural fractures in the model, the cumulative oil and gas production increase by 1.5% and 6.8%, respectively, compared to the non-planar case. As 1,000 natural fractures exist in one set in the model, the cumulative oil and gas production is 2.8% and 11.4% higher, respectively. If 1,000 natural fractures set in two sets, the cumulative oil and gas production can be as high as 5.0% and 14.0%, respectively, reflecting a significant impact. That is, complex fracture geometries have a positive influence on the well performance in tight reservoirs. When the number of natural fractures in one set increases from 500 to 1,000, more natural fractures connect to hydraulic fractures, leading to the increase in the cumulative production. When 1,000 natural fractures change from one set to two sets, it can be found that there are more natural fractures connecting to hydraulic fractures and also more natural fractures connecting each other. Figure 4 depicts the difference of pressure distribution. As shown, the more complex the fracture geometries are, the more overlaps between the neighboring wells. The pressure of Case 4 is closer to the minimum value (color is closer to purple). The difference of average pressure of four cases is plotted in Fig. 4(e) to better present the pressure drawdown in a 30-year period. It can be seen that the pressure decreases faster when the fracture geometries become complex, especially at the beginning of production time (5 years). In addition, the difference of pressure distribution for four cases in Fig. 4 shows that the drainage area becomes larger as the fracture geometries become complex. Hence, the increase in the cumulative production is more obvious. The fluid flows more easily from the formation to the wellbore due to the more connected networks.

The oil saturation of the four cases in the production period is presented in Supplementary Figure 2. The noticeable change in the oil saturation indicates that the presence of natural fractures provides more connected fracture networks. The high conductivity allows the oil and gas to transport more easily from matrix to the horizontal wells with higher transmissibility and improves the well performance. Hence, the natural fractures should be included in the simulation model in tight formations, especially with high density of natural fractures.

### Effect of nanopore confinement on well performance for different fracture geometries

**Figure 5:**
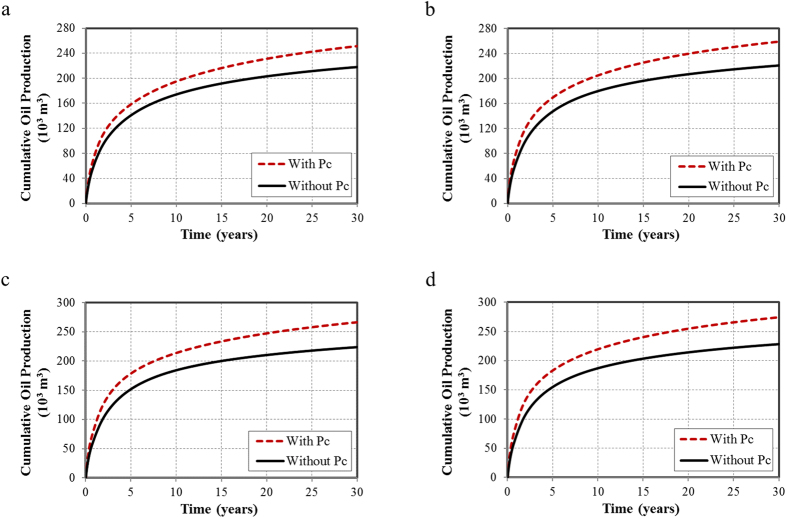
Cumulative oil production for a 30-year period with and without the capillary pressure effect. (**a**) Non-planar hydraulic fractures; (**b**) Non-planar hydraulic fractures with 500 NF in one set; (**c**) Non-planar hydraulic fractures with 1,000 NF in one set; (**d**) Non-planar hydraulic fractures with 1,000 NF in two sets.

**Figure 6:**
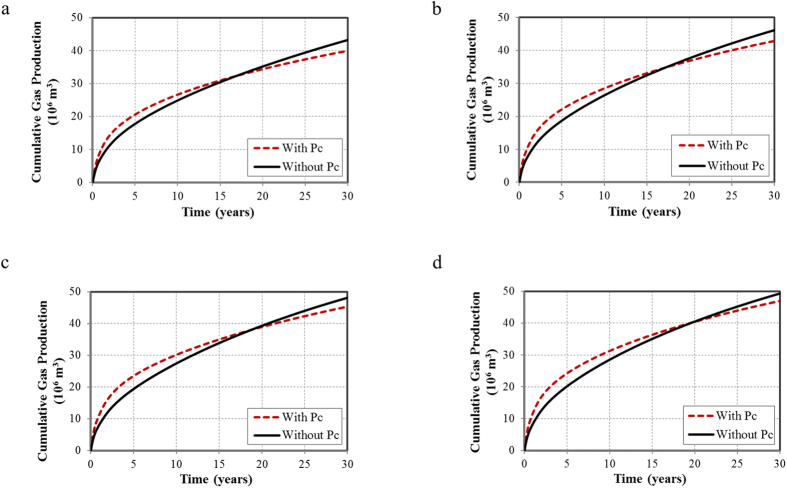
Cumulative gas production for a 30-year period with and without the capillary pressure effect. (**a**) Non-planar hydraulic fractures; (**b**) Non-planar hydraulic fractures with 500 NF in one set; (**c**) Non-planar hydraulic fractures with 1,000 NF in one set; (**d**) Non-planar hydraulic fractures with 1,000 NF in two sets.

In this section, we evaluated the capillary pressure effect with the aforementioned fracture geometries. The cumulative oil and gas production for a 30-year period with and without the capillary pressure effect are summarized in Figs 5 and 6, respectively.

**Figure 7:**
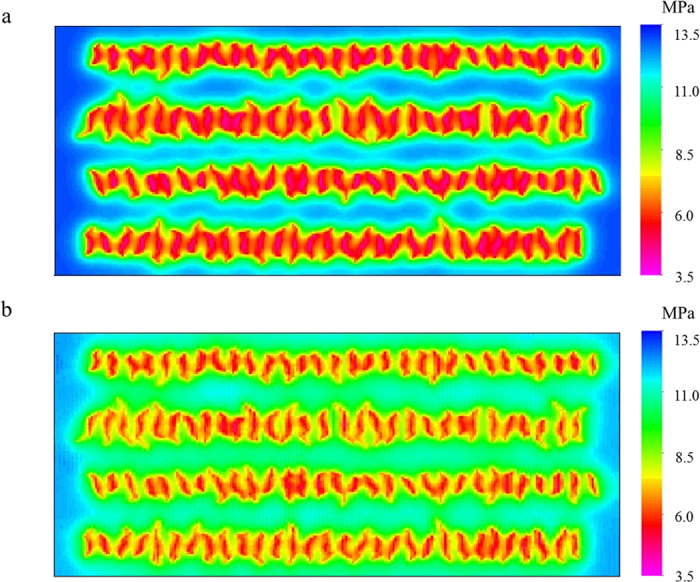
Pressure distribution of the non-planar fractures case at the end of 30-year production with and without the capillary pressure effect. (**a**) Without the capillary pressure effect; (**b**) With the capillary pressure effect.

The cumulative oil productions in Fig. 5 increase when the capillary pressure effect is taken into account. As a result of the bubble-point pressure decreases, the single-phase production period becomes longer and it improves the oil production. In comparison to the oil production in a period of 30 years for each fracture geometry, the cumulative oil productions with the capillary pressure effect are 15%, 17.5%, 19%, and 20% higher than the cases without the capillary pressure effect. The difference of pressure distribution clearly describes the capillary pressure effect. Only non-planar fractures case is presented for neatness in Fig. 7. As illustrated, pressure drops rapidly and drainage area becomes larger when the capillary pressure effect is taken into account, leading to the increase in the cumulative oil production at the end of 30 years.

The capillary pressure effect for cumulative gas production is more complex than oil (Fig. 6). In the early period, the gas production of the case with the capillary pressure effect is higher compared to the case without the capillary pressure effect. The reason being that when considering the capillary pressure effect, more oil is produced with solution gas. The solution gas is released and dominates gas production at the beginning. As the reservoir pressure depletes, it will reach the bubble-point pressure; the fluid goes from single-phase to two-phase region and gas production continues to increase. In this period, solution gas does not dominate the gas production any longer. It can be observed that the bubble-point pressure reduces by 1.4 MPa at the reservoir temperature of 116 °C due to the capillary pressure effect (Supplementary Figure 3). The reduction caused a shorter two-phase period for the case with the capillary pressure effect, leading to less gas production in the later period. Additionally, the intersection time between the cases with and without the capillary pressure effect will move to a later time when the fracture geometries become more complex.

### Effect of matrix permeability on well performance

Matrix permeability is another key parameter affecting the well performance in tight oil reservoirs. In this section, the permeability value of 0.1 md is selected to evaluate the well performance for different fracture geometries. The capillary pressure effect is taken into account and the results were compared with the case of 0.01 md.

**Figure 8:**
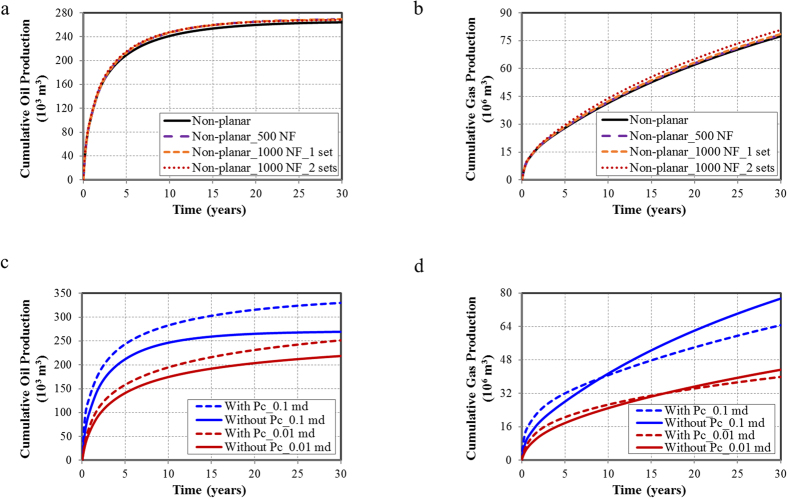
Well performance for a 30-year period with the permeability of 0.1 md. (**a**) Cumulative oil production for a 30-year period for different fracture geometries with the permeability of 0.1 md; (**b**) Cumulative gas production for a 30-year period for different fracture geometries with the permeability of 0.1 md; (**c**) Cumulative oil production of the non-planar fractures case with the permeability of 0.01 md (blue curves) and 0.1 md (red curves); (**d**) Cumulative gas production of the non-planar fractures case with the permeability of 0.01 md (blue curves) and 0.1 md (red curves).

Figure 8(a) and (b) present the cumulative oil and gas production with different fracture geometries under the permeability of 0.1 md. Compared to the non-planar hydraulic fractures case, the cumulative oil production increases 0.8% for the non-planar hydraulic fractures with 500 NF case, 1.5% for the non-planar hydraulic fractures with 1,000 NF in one set case, and 2.1% for the non-planar hydraulic fractures with 1,000 NF in two sets case. The cumulative gas production increases 1.3%, 3.0%, and 4.3%, respectively. The incremental oil and gas production reflect a smaller impact compared with the results in Fig. 3. That is, the presence of natural fractures plays a less significant role in the case of high matrix permeability.

The well performance of the non-planar fractures case is analyzed in detail, as shown in Fig. 8(c) and (d). In comparison with the results with matrix permeability of 0.01 md, the cumulative oil production increases by 24% for high permeability (blue and red solid lines). The larger incremental oil production is observed in Fig. 8(c) when the capillary pressure effect is considered (dash line and solid line), indicating that the capillary pressure effect is more significant for high permeability. For cumulative gas production in Fig. 8(d), the similar results can be observed.

The differences of the average pressure and oil saturation between low and high permeability cases without the capillary pressure are presented in Supplementary Figures 4 and 5. The pressure drops 3.1 MPa higher and oil saturation decreases 10% higher for the high permeability case than the low permeability case. The large difference indicates the increase in the production of high permeability case (0.01 md).

Although high permeability represents larger pore sizes, which is assigned to small capillary pressure, a small difference with and without capillary pressure for the high permeability is expected. However, in this study, it is assumed that the capillary pressure is independent of pore sizes and is constant for both permeability values. Consequently, a high difference with and without capillary for the high permeability case is seen. The relationship between permeability and pore sizes is beyond the scope of this work, which will be investigated in future works.

## Discussion

In this study, the effects of various fracture geometries and capillary pressure on multi-well performance are investigated. The EDFM approach is applied to describe the effect of fluid flow in the complex fracture geometries. The presence of natural fractures contributes to the multi-well performance; the incremental oil and gas production of the non-planar hydraulic fractures case with 1,000 NF in two sets is 5% and 14% higher compared to the non-planar hydraulic fractures case.

Based on the well performance for different fracture geometries with and without the capillary pressure effect, the cumulative oil production increases when considering the capillary pressure effect. However, the incremental oil production is similar for various fracture geometries. The gas production, in the early period, is higher compared to the case without the capillary pressure effect due to the solution gas; however, the gas production of the case without capillary pressure improves because of longer two-phase period. Additionally, the intersection time between cases with and without the capillary pressure effect moves to a later time for complex fracture geometries.

For the cases with high permeability of 0.1 md, the incremental oil and gas productions for the non-planar hydraulic fractures with 1,000 NF in two sets are 2.1% and 4.3%, respectively, which indicates the contribution of natural fractures is less significant for high permeability. Therefore, the natural fractures should be included in the numerical model of tight formations, especially for low permeability and high density of natural fracture networks.

### Methodology

#### Black-oil fluid properties affected by nanopore confinement

Flash calculation accounting for the inequalities of liquid and vapor phases is applied to calculate the fluid properties, *i*.*e*. oil and gas viscosities, densities, solution gas-oil ratio as a function of oil pressure. In this work, the oil pressure is assumed as the refrence pressure. Hence, the gas pressure is calculated using the Young-Laplace equation^28^ given below:


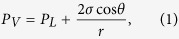


where *P*_*V*_ and *P*_*L*_ refer to the pressures in the vapor and liquid phase, respectively. *r* is the radius of capillary tube in porous media; *θ* is the contact angle representing the surface tension between solid and fluids; *σ* is the interfacial tension at the vapor-liquid phase interface.

Phase equilibrium is achieved when the fugacities of components in the vapor and liquid phases are equal^29^. The criterion is given as


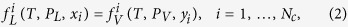


where 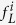 and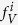 represent the fugacity of component *i* in the liquid and vapor phases. *T* is the reservoir temperature; *N*_*c*_ is the number of fluid components. *x*_*i*_ and *y*_*i*_ are liquid and vapor compositions, respectively.

Peng-Robinson equation of state and Rachford-Rice flash calculation are modified considering the difference between the vapor and liquid pressures. The mass balance equations are applied as well, which are given in Equations (3) through (5).


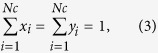



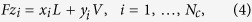



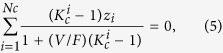


where *z*_*i*_ is the overall mole fraction of component *i. F* is the number of moles of original feed. *L* and *V* are the number of moles of liquid and vapor phases, respectively. 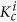 is the *K*-values considering the inequalities between liquid and vapor pressures. More details about the phase equilibrium calculation can be found in the work by Zhang *et al*.^21^.

Based on phase equilibrium calculation, the black-oil fluid properties such as viscosities, solution gas-oil ratio (*R*_*s*_), and formation volume factor (*B*_*o*_) is determined in the Middle Bakken formation for different pore sizes. In order to obtain the solution gas-oil ratio, *R*_*s*_, the equilibrium liquid composition is flashed at the standard conditions. The equation is described as


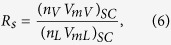


where *n*_*V*_ and *n*_*L*_ are the vapor and liquid phase molar fraction, respectively. *V*_*mV*_ and *V*_*mL*_ are the vapor and liquid molar volume, respectively. *SC* represents the standard condition.

The oil formation volume factor *B*_*o*_ is defined as


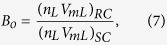


where *RC* represents the reservoir condition.

The oil viscosity is calculated by^30^


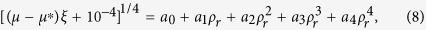


where *μ* is the oil viscosity at the reservior condition; *a*_*0*_ through *a*_*4*_ are 0.1023, 0.023364, 0.058533, −0.040758 and 0.0093324, respectively. *ρ*_*r*_ is the reduced density, which is defined as


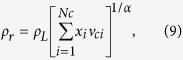


where *v*_*ci*_ is identical to the critical volume of component *i*; the value of parameter *α* is equal to 1.

The mixture viscosity parameter *ξ* is evaluated as


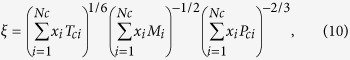


where *M*_*i*_ is the molecular weight of component *i*.

The low pressure viscosities of the mixture in Equation (8) is defined as


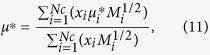


where 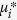, the low pressure viscosities for pure substances are calculated using Stiel and Thodos equation^31^:


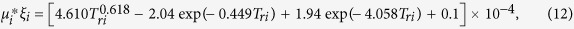


with 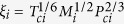 and 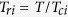.

The calculation of the oil viscosity also can be applied in the gas viscosity. We note that the properties calculation discussed above are not limited to the fluid properties considering the capillary equilibrium. Once the capillary pressure is set to zero, it can then be applied to the bulk fluid. The tuned PVT data is input into a standard black-oil simulator to predict the well performance in the Middle Bakken formation.

### Embedded Discrete Fracture Model (EDFM)

The complex fracture geometries in tight oil reservoirs are common because of the interaction of hydraulic fractures and the pre-existing natural fractures. It is important to accurately model the influence of complex fractures on well performance in reservoir simulation. Traditional LGR approach can accurately model the fluid transport from matrix to fractures, but it is difficult to deal with complex non-planar fractures^32^. The EDFM is an efficient approach to handle the complex fracture geometries through discretizing the fractures into segments with matrix cell boundaries^33^,^34^,^35^. In addition, virtual cells are added for these fracture segments. The Non-Neighboring Connections (NNCs) are used for these cells to account for fluid transport associated with fractures, including the flow between matrix and fractures, flow inside an individual fracture, and flow between intersecting fractures. The volume flow rate of phase *l* between cells in a NNC pair is


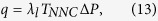


where *λ*_*l*_ is the relative mobility of phase *l*, Δ*P* is the potential difference between the cells, and *T*_*NNC*_ is the NNC transmissibility factor, which can be calculated by


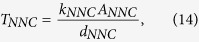


where *k*_*NNC*_, *A*_*NNC*_, and *d*_*NNC*_ represent the permeability, contact area, and distance associated with this connection, respectively. For the matrix-fracture connection, *k*_*NNC*_ is the matrix permeability in the direction perpendicular to the fracture plane, *A*_*NNC*_ is the area of the fracture plane inside the matrix block, and *d*_*NNC*_ is the average normal distance from matrix block to fracture plane. For the connections between fracture segments, *k*_*NNC*_ is an average of fracture permeability, *A*_*NNC*_ is the common area between fracture segments, and *d*_*NNC*_ is the distance between centroids of the fracture segments. For the fracture-wellbore intersections, an effective well index is used and calculated by


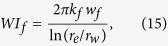



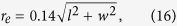


where *k*_*f*_ is the fracture permeability, *w*_*f*_ is the fracture aperture, and *l* and *w* are the fracture segment length and height, respectively. More details about the calculations of NNC transmissibility factors can be found in the work by Xu *et al*.^26^. In addition, the EDFM approach can be applied in traditional reservoir simulators in a non-intrusive manner^26^.

## Additional Information

**How to cite this article**: Zhang, Y. *et al*. A Comprehensive Numerical Model for Simulating Fluid Transport in Nanopores. *Sci. Rep.* **7**, 40507; doi: 10.1038/srep40507 (2017).

**Publisher's note:** Springer Nature remains neutral with regard to jurisdictional claims in published maps and institutional affiliations.

## Supplementary Material

Supplementary Information
