# Supplementary material for: A Comprehensive Numerical Model for Simulating Fluid Transport in Nanopores
Source: Sci Rep. 2017 Jan 16;7:40507. doi: 10.1038/srep40507 (PMC5238443; doi:10.1038/srep40507)
Supplement: Supplementary Information [file srep40507-s1.pdf]

Supplementary information to:

A Comprehensive Numerical Model for Simulating Fluid Transport in Nanopores

Authors: Yuan Zhang[1], Wei Yu[2,+], Kamy Sepehrnoori[3], Yuan Di[1,+]

Affiliations: [1]College of Engineering, Peking University, Beijing, 100871, China.

[2]Department of Petroleum Engineering, Texas A & M University, Collage Station, TX, 77843, USA.

[3]Department of Petroleum and Geosystems Engineering, University of Texas at Austin, Austin, TX, 78712, USA.

[+]Corresponding author

Contact Information of Corresponding Authors:

Yuan Di, Ph.D., College of Engineering, Peking University, 5, Yiheyuan Road, Beijing, 100871, China. Tel: +86-135-5270-9129. E-mail: diyuanmech@126.com.

Wei Yu, Ph.D., Department of Petroleum Engineering, Texas A & M University, Collage Station, TX, 77843, USA. Tel: +1-512-574-0080. E-mail: yuwei127@gmail.com.

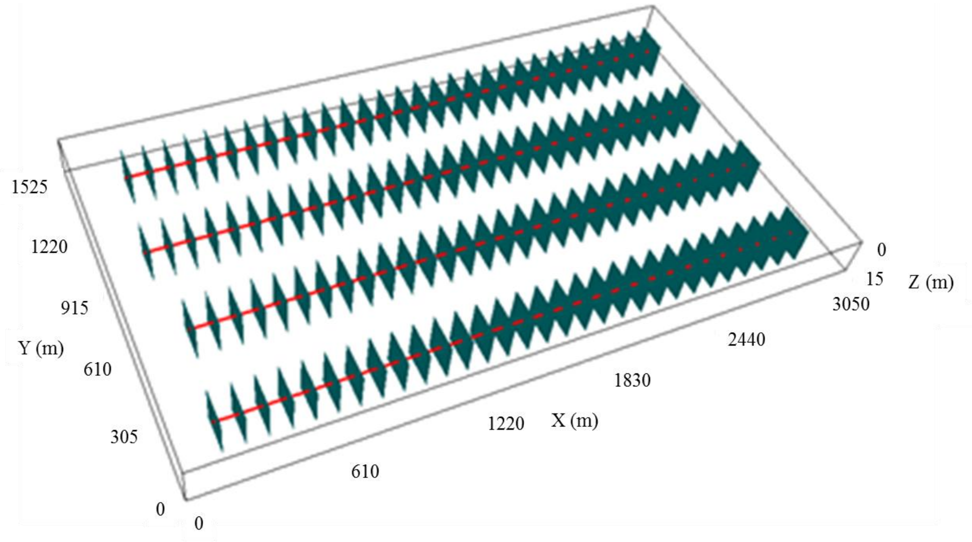

Supplementary Figure 1: A base reservoir model including four horizontal wells with 30 planar hydraulic fractures for each well.

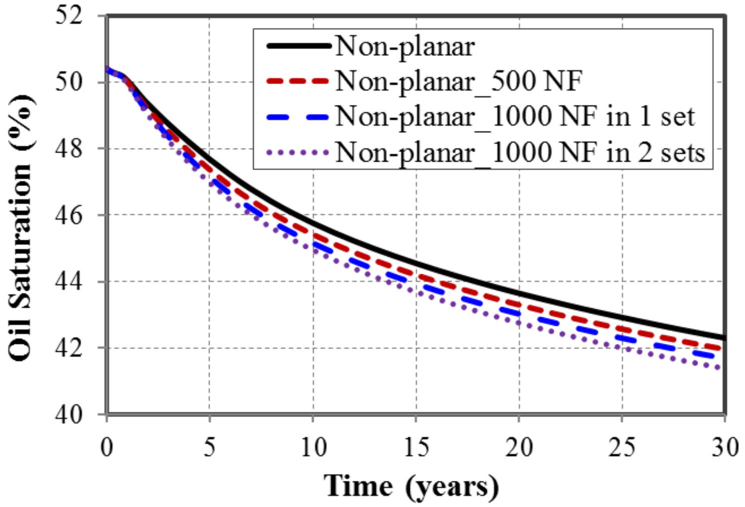

Supplementary Figure 2: Oil saturation for four different cases in a 30-year period.

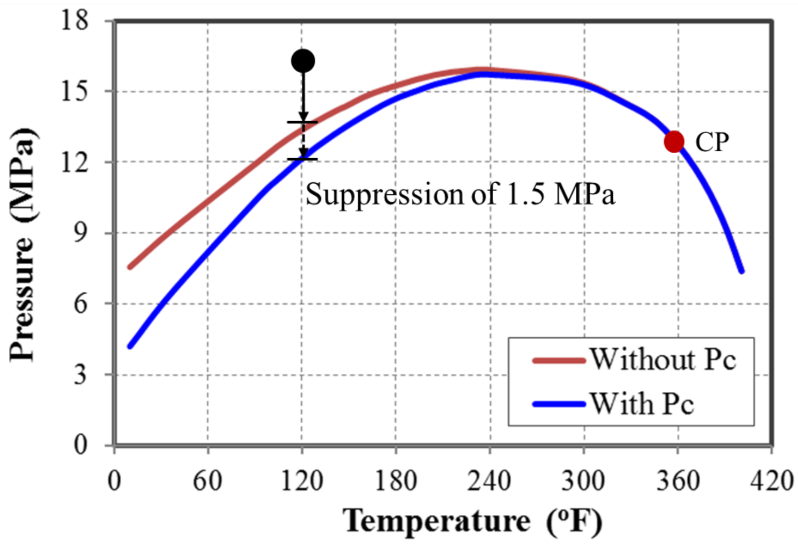

Supplementary Figure 3: Phase envelope for the Middle Bakken fluid at different pressures and temperatures.

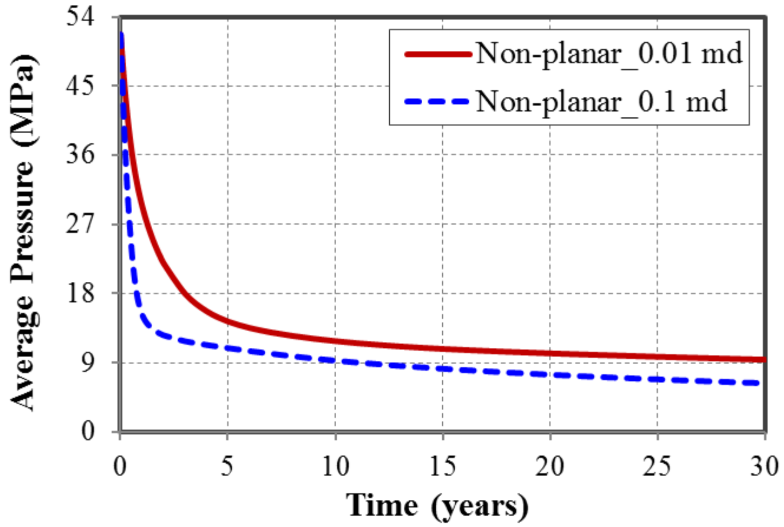

Supplementary Figure 4: Comparison of average pressure for different permeability cases.

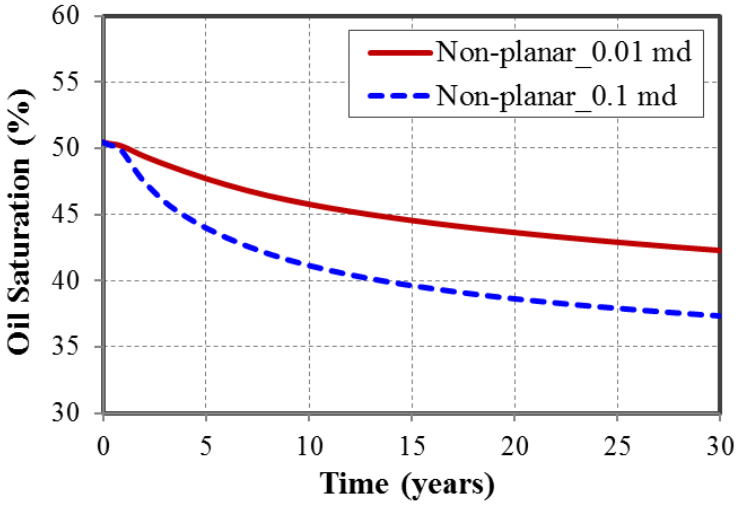

Supplementary Figure 5: Comparison of oil saturation for different permeability cases.

Calculation of cumulative production:

Cumulative production of oil and gas can be calculated by multiplying the amount of production by the rate. Suppose the well's production rate starts to decline when a critical (lowest permissible) bottomhole pressure is reached. The production rate at a given decline time t can be expressed as:

$$q = \frac{kh(\bar{p}_t - p_{wf})}{141.2 B_o \mu [\ln\left(\frac{0.472 r_e}{r_w}\right) + s]}, \quad \text{(S-1)}$$

where k represents the permeability, h is the reservoir height, B_o is the formation volume factor, μ is the fluid viscosity, $\bar{p}_t$ is the average reservoir pressure at decline time t, p_{wf} is the bottomhole pressure maintained during the production decline.

The cumulative oil production of the well after the production decline time t can be expressed as:

$$N_p = \int_0^t \frac{kh(\bar{p}_t - p_{wf})}{141.2 B_o \mu [\ln\left(\frac{0.472 r_e}{r_w}\right) + s]} dt \,. \quad \text{(S-2)}$$

It is based on the mass balance equation, which is defined as:

$$\begin{Bmatrix} \text{rate of} \\ \text{accumulation} \\ \text{of } i \text{ in } V \end{Bmatrix} = \begin{Bmatrix} \text{Net rate of } i \\ \text{transported} \\ \text{into } V \end{Bmatrix} + \begin{Bmatrix} \text{Net rate of} \\ \text{production} \\ \text{of } i \text{ in } V \end{Bmatrix}. \tag{S-3}$$

The calculation for each term has been explained in the previous work (Lake, 1989).

References:

Lake, Larry W. Enhanced oil recovery. Prentice Hall, (1989).
